# Celebrity Endorsement, Brand Equity, and Green Cosmetics Purchase Intention Among Chinese Youth

**DOI:** 10.3389/fpsyg.2022.860177

**Published:** 2022-03-07

**Authors:** Zhai Lili, Abdullah Al Mamun, Naeem Hayat, Anas A. Salamah, Qing Yang, Mohd Helmi Ali

**Affiliations:** ^1^UCSI Graduate Business School, UCSI University, Kuala Lumpur, Malaysia; ^2^UKM – Graduate School of Business, Universiti Kebangsaan Malaysia, UKM Bangi, Selangor, Malaysia; ^3^Faculty of Entrepreneurship and Business, Universiti Malaysia Kelantan, Kota Baharu, Malaysia; ^4^College of Business Administration, Prince Sattam Bin Abdulaziz University, Al-Kharj, Saudi Arabia

**Keywords:** celebrity endorsement, brand equity, attitude, purchase intention, green cosmetics, Chinese youth

## Abstract

The study examined the effect of celebrity attractiveness, celebrity trustworthiness, and celebrity cause fit on the attitude toward green cosmetics. This was followed by the effect of brand awareness, brand associations, brand loyalty, perceived quality, brand credibility on brand equity, including the impact of attitude toward green cosmetics and brand equity on the willingness to purchase green cosmetics among of young Chinese consumers. This study adopted a cross-sectional design and collected quantitative data from 301 respondents using a structured questionnaire, which was distributed online using various social media platforms. It was found that celebrity attractiveness, celebrity trustworthiness, and celebrity cause-fit had a significant impact on the attitudes toward green cosmetic, while brand loyalty, perceived quality, and brand credibility substantially affected brand equity. Moreover, the attitudes toward green cosmetics and brand equity had a strong impact on the willingness to purchase green cosmetics. To increase the sales for green cosmetics, the advertisements for it should have appeal, trustworthiness, and cause-fit celebrities to improve consumers’ attitudes and willingness to purchase green cosmetics. Finding of this study provide a guideline for green cosmetic manufacturers, to direct their resources to enhance brand loyalty, credibility, and perceived quality of the product they produce by highlighting the difference between conventional and green cosmetics.

## Introduction

Significant changes in people’s lives have taken place with the progress of social science and technology. However, despite the convenience and comfort of the social changes from science and technology, severe environmental problems could also occur (Nam et al., 2017). The deterioration of the environment is gradually threatening the ecological balance of nature. Therefore, environmental protection has become the world trend, while green consumption advocates production and consumption activities based on the coordinated development of nature (Akturan, 2018). Studies demonstrated that most consumers were willing to pay higher prices for green products and were more inclined to choose products with a green logo when selecting similar products (Beatson et al., 2020).

Two aspects defined green products. Specifically, the products could be represented through the production process to reduce non-renewable resources and environmental pollution (Nam et al., 2017). Another aspect is indicated through product performance (Beatson et al., 2020). To exemplify accurately, the product ingredients do not contain chemicals polluting the environment, while the green and environment-friendly ingredients are adopted. Green cosmetics mainly refer to cosmetics made with purely natural raw materials as the main ingredients (Umberson, 2008). However, no other flavors and preservatives are added in the production process to reduce the harm of chemical synthesis to the environment and the human body (Sharma and Foropon, 2019). With the continuous improvement in consumers’ awareness of green and environmental protection, the demand for cosmetics in the Chinese market has also changed. Besides, green consumption has become a mainstream trend (Beatson et al., 2020). With the gradual improvement in living standards, people show more willingness to pay for healthy and natural products (Nam et al., 2017). Overall, these objective conditions directly promote the development of green cosmetics. Consumers tend to pay more attention to cosmetics with higher health and safety attributes (Umberson, 2008). To illustrate, environment-oriented consumers select products that contain natural ingredients and no preservatives (Sharma and Foropon, 2019).

Since 2009, China’s cosmetics industry has been emphasizing environmental protection. Various enterprises are actively performing research and development, which develops a sense of social responsibility and advocates the adoption of natural ingredients and using environmental protection materials to reduce environmental pollution. A deeper understanding of Chinese consumers’ attitudes helps promote green cosmetic consumption in China. The spread of globalization promotes the development of trade and the diversity of commodities, with the continuous diversification of categories and demands of consumers (Sikder et al., 2019). As business opportunities are created in various industries, manufacturers are faced with tremendous pressure to produce products according to the consumers’ needs (Akturan, 2018). With the development of the global economy, resources are strained, while environmental pollution increases and leads to other issues in people’s daily lives (Sikder et al., 2019). This situation leads to changes in the consumption structure as the level of consumption has continuously improved. People have begun to pay more attention to health, apply the scientific way of consumption, and gradually strengthen green purchase awareness (Beatson et al., 2020).

The continuous development of the global economy leads to a tremendous increase in income levels. Besides the essential daily expenses, people begin to pay more attention to other products to improve life quality. For example, modern young women pay more attention to skincare and dressing, promoting the cosmetics industry’s continuous development (Al-Mamun et al., 2020). Given that the cosmetics industry is fast-paced, guaranteeing customer loyalty in today’s era is not a simple challenge (Sikder et al., 2019). Consumers are more attentive toward product quality and willing to pay for the quality (Beatson et al., 2020). With the changing consumers’ demands, young consumers prefer to consume green products as a fashion (Al-Mamun et al., 2020). The increased attention toward health and healthcare directly promotes the demand for natural food cosmetics (Sikder et al., 2019).

The effect of celebrity endorsement and brand equity is crucial for understanding market demand (Ilicic and Baxter, 2014). The celebrity endorsement impacts the consumers’ attitude (Patel and Basil, 2017). However, celebrity endorsement influences the attitude with its three dimensions: attractiveness, trustworthiness, and cause-fitness (Tantawi and Sadek, 2019). For the current study, the separation of celebrity endorsement in three dimensions helps to fully expose the celebrity brand endorsement nurturing the brand attitude. Brand personality has become a significant characteristic of products, in which charging the consumer memory and developing purchase intention are the vital aspects of today’s marketing management (Ozdemir et al., 2020). Celebrity endorsement and brand value are significant for developing brand equity among consumers. The current work aims to explore celebrity endorsement from its attractiveness, trustworthiness, and cause fit affecting the brand awareness, brand association, brand loyalty, perceived quality, and brand credibility, which lead to brand equity and willingness to purchase green cosmetics among young Chinese consumers.

## Literature Review

### Celebrity Endorsement and Attitude Toward Green Cosmetics

Celebrity defines a particular type of famous person recognized by consumers (Rifon et al., 2015). These celebrity figures have a unique personality or lifestyle, separating them from ordinary people (Roll, 2015). Furthermore, they often have high social popularity and are classified according to their achievements in various fields (Patel and Basil, 2017). Celebrity endorsement brings particular influence to the product, while the brand information transmitted by celebrities is often more convincing compared to the information conveyed by non-celebrities (Stone et al., 2014). Consumers are more likely to buy products endorsed by their favorite celebrities. This research separated celebrity endorsement into three parts: celebrity attractiveness, celebrity trustworthiness, and celebrity cause fit.

#### Celebrity Attractiveness

The endorsement of celebrities or influential may affect consumers’ attitudes toward products or services (Rifon et al., 2015). Although some celebrities are not famous stars or athletes, their endorsements remain attractive as long as consumers are willing to recognize their talents or appearance (Belch and Belch, 2018). Consumers may be deeply impressed by a particular product due to the celebrities’ appeal or change their previous views or attitudes toward a specific product when this appeal varies (Hoegele et al., 2015). Physically attractive celebrities are regarded as having more marketing advantages than the opposite. Although celebrities do not have the same level of fame as industry stars, audiences appreciate their appearance, words, or behavior as long as they possess a particular attractiveness (Arora et al., 2019). The higher attractiveness of the celebrity contributes to higher sales of the products being endorsed, leading to increased profits of enterprises (Roll, 2015). Celebrity attraction is an essential factor influencing consumers’ attitudes toward green cosmetics (Tantawi and Sadek, 2019).

Celebrities are attractive in various ways, such as being inspirational or elegant, intelligent, or beautiful (Hoegele et al., 2015). The higher the attractiveness of the celebrity endorsement, the image of the product or service would receive higher recognition. When an attractive celebrity promotes a company, consumers will often be influenced and present approval. As a result, consumers positively respond to relevant products (Arora et al., 2019). Based on the conclusion from the previous literature, this study hypothesized that the attractiveness of celebrity endorsement would have a positive impact on consumers’ attitudes toward products.

Hypothesis (H1): *Celebrity attractiveness has a positive impact on attitude toward green cosmetics*

#### Celebrity Trustworthiness

The credibility of celebrity endorsements indicates that the celebrity is honest, has professional ethics, and is trusted by consumers (Hoegele et al., 2015). The most trustworthy stars are unwilling to endorse unreliable products despite the temptation of financial interests (Belch and Belch, 2018). This situation possibly leads to a negative impact on their reputation and word of mouth. When consumers trust a product spokesperson, the celebrity would be influenced to change their product acceptance (Hoegele et al., 2015).

The higher credibility of the celebrity leads to a more persuasive advertisement. This feature will also increase consumers’ trust in products and gain their attention. Notably, celebrity endorsement is proven to be an effective marketing method (Ilicic and Baxter, 2014). Regardless of the type of enterprises and organizations, consumers make the utmost effort to find highly reliable celebrities as spokespersons (Yoo et al., 2018). The reliability of celebrities would affect consumers’ acceptability to consume a product (Wymer and Drollinger, 2014). For example, charities often need to find reliable celebrity endorsements due to consumers’ expectations that such advertisements should be non-deceptive and reliable. As a result, charities tend to increase money directly proportional to celebrity endorsements’ reliability (Thamaraiselvan et al., 2017).

Wymer and Drollinger (2014) demonstrated that celebrity attraction is an essential factor influencing consumers’ attitudes toward audience donation intentions. Meanwhile, Tantawi and Sadek (2019) confirmed a positive relationship between celebrity trustworthiness and purchasing attitude among consumers for cause-related marketing. Considering the above-documented literature, it was assumed that celebrities’ trust degree was directly proportional to consumers’ attitudes toward products.

Hypothesis (H2): *Celebrity trustworthiness positively impacts attitudes toward Green Cosmetics.*

#### Celebrity Cause-Fit

Celebrity endorsement works well when the celebrity fits with the message celebrity endorsing; the match between the product characteristics, celebrity, and brand fitness instigates the consumer buying behavior (Yoo et al., 2018). A good fit between celebrities and product nature increases the advertisements’ persuasive power and publicity effectiveness (Kim and Na, 2007). Higher compatibility between the celebrity and brand leads to a more persuasive advertisement (Kim and Na, 2007). The brand image should match the spokesperson’s image, i.e., celebrity (Knoll and Matthes, 2016). To illustrate, sports brand advertisement must invite famous athletes as a celebrity. In contrast, the educational institutions should invite celebrities with an excellent reputation to promote the education services. Similarly, shampoo advertisements should invite the suitable fashion icon having the appropriate images as to represent a brand as spokespersons, and product advertisers always look for a match between the product and celebrity, celebrity fitness significantly influences the attitude toward advertised products (Ilicic and Baxter, 2014).

Thamaraiselvan et al. (2017) confirmed a positive relationship between celebrity cause fitness and purchasing attitude. Therefore, a precise fit is a must between celebrity endorsement and brand products to promote consumers’ positive influence on consumers’ minds. Ilicic and Baxter (2014) postulated that celebrity brand fit is an essential factor influencing consumers’ attitudes toward green cosmetics. Hoegele et al. (2015) postulated that a substantial relationship between celebrities and products would lead to consumers’ positive comments. The celebrity cause-fitness with the product endorsement influences the prospective consumer’s attitude to buy fashion consumables (Ilicic and Baxter, 2014). Therefore, the current work expected the following hypothesis:

Hypothesis (H3): *Celebrity cause-fit has a positive impact on attitude toward green cosmetics*

### Brand Equity

Brand equity consists of brand loyalty, perceived quality, brand awareness, brand relevance degree, and other dimensions in many studies. These factors present the enterprise brand equity in the market value.

#### Brand Awareness

Brand awareness refers to the degree of recognition of a brand or product in consumers’ minds and is an integral part of brand equity (Sharma, 2017). Different brands have different brand images and characteristics. This unique brand positioning in consumer minds is known as brand awareness, which affects consumers’ brand awareness and equity. Celebrity attraction is an essential factor influencing consumers’ attitudes toward green cosmetics (Sharma and Foropon, 2019). Rossiter’s (2014) study confirmed the positive relationship between celebrity attraction and purchasing attitude.

Brand awareness mainly comprises two parts: consumers’ cognition of the brand and the consumers’ memory of the brand. Higher brand awareness facilitates consumers in gaining an impression of the brand, choosing certain types of goods, and prioritizing their familiar brands (Sharma, 2017). Brand awareness could help consumers remember a particular brand. Therefore, consumers could exclude other brands from the same category and form a brand effect (Pappu and Quester, 2015). Given that consumers tend to buy their familiar brands, brand awareness through ascension would directly affect brand value and brand assets (Gui, 2015). We like to propose the following:

Hypothesis (H4): *Brand awareness has a positive effect on brand equity*

#### Perceived Quality

Perceived quality is the consumer’s perception of the product or service based on its subjective judgment instead of its inherent characteristics or service. The perceived quality of a brand could affect consumers’ acceptance of their purchased goods (Sarkar et al., 2015). The core of perceived value is the trade-off between perceived benefits obtained by customers and paid costs, while the perceived value is customers’ evaluation of product attributes and effects (Pappu and Quester, 2015). When the perceived quality is higher than the consumers’ original expectations, consumers will purchase the product (Rifon et al., 2015; Rizvi et al., 2018). Ramaseshan and Tsao highlighted in their research that a brand perceived quality plays a critical role in enterprises and brand growth and development (Till et al., 2011). Products with fashionable styles or characteristics will provide consumers with a higher quality perception experience, attracting more consumers.

The brand’s perceived value affects consumers’ choice or exclusion of the brand, which is also an essential factor of brand equity (Sarkar et al., 2015). With higher perceived quality, a better premium ability could benefit the brand. Notably, brand perceived quality is an essential factor influencing brand equity (Park et al., 2019). Sharma and Foropon (2019) confirmed the positive relationship between perceived quality and brand equity. In this case, the brand scholarship illustrated that perceived quality is an integral part of brand equity thus consumers will choose specific brands for products with high perceived quality (Park et al., 2019). Subsequently, consumers’ perceived quality would improve, followed by increased brand equity (Kieu, 2019). The following hypothesis was presented:

Hypothesis (H5): *Perceived quality has a positive effect on brand equity*

#### Brand Loyalty

Brand loyalty is a type of repeated purchase behavior, which represents that consumers would constantly purchase a specific brand or a few fixed brands (Andonova et al., 2014). It was proven from empirical literature that enterprises with more focus on building brand loyalty would gain higher profit returns and a larger market share (Belch and Belch, 2018). Brand satisfaction depends on consumers’ purchase experience, while good products or services would form customer retention (Kajalo and Jyrämä, 2016). Enterprises’ commitment to improving brand loyalty contributes to additional revenue and profits. At the same time, other unnecessary expenses are reduced to emphasize maintaining existing customers and developing future customers (Ozdemir et al., 2020). Brand loyalty indicates consumers’ willingness to buy products of specific brands without being influenced by competitors of other brands in the future (Pappu and Quester, 2015). Product satisfaction contributes to brand loyalty when it reaches a particular condition, given that higher brand loyalty increases the probability of product buyback (Keller, 2018). The repurchase rate brings profits to the enterprise, increases its value, and forms brand assets (Knoll and Matthes, 2016). Brand loyalty is an essential factor influencing brand equity (Akturan, 2018), while brand loyalty contributes to repurchase intention (Kajalo and Jyrämä, 2016). Therefore, consumers’ willingness to repurchase transforms into the sales volume of the enterprises that improve brand assets (Pappu and Quester, 2015). The increase of brand equity depends on consumers’ loyalty to the brand. Overall, this study assumed that brand loyalty positively impacted brand equity.

Hypothesis (H6): *Brand loyalty has a positive effect on brand equity*

#### Brand Associations

Brand association refers to the brand information in the consumers’ memory (Keller, 1993). Different brands have different degrees of association among consumers. It was found that brand personality would create different sensory experiences for consumers, leading to various brand associations (Maheshwari et al., 2016). Therefore, brand memory points toward the essential aspect of brand equity (Rossiter, 2014). The brand association could be related to the enterprise product or its product (Keller, 1993). For example, people often combine coke, Starbucks, and other products with young consumer groups (Keller, 2016). If the brand association is in line with the consumers’ personality upon purchasing a particular brand of goods, the consumer will be inclined to buy a specific brand (Keller, 1993). To illustrate accordingly, boys interested in basketball would buy Jordan’s shoes as this choice indicates their preferences and personality (Yoo et al., 2018). Till et al. (2011) suggested that when brand association resonates with consumers’ emotions, the brand could form its status and image in the market, positively impacting brand equity.

The brand association creates a positive influence on consumers’ hearts and creates brand value by forming personalized images (Yoo et al., 2018). Consumers express their personality and characteristics by purchasing certain goods, which indicates that the brand association and personality present personal meaning to the consumers (Tantawi and Sadek, 2019). The marketing purpose of green products is to form a functional or emotional brand association, which is reflected in the brand association to convey the attributes of green products, while the emotional aspect is to develop an emotional resonance with consumers through the awareness of environmental protection (Andonova et al., 2014). Based on the above literature, the brand association is the foundation of brand equity and will impact consumers’ consumption behavior. Therefore, brand association is the source of brand equity.

Hypothesis (H7): *Brand association has a positive effect on brand equity*

#### Brand Credibility

Brand credibility refers to the trustworthiness of product information in a brand (Akturan, 2018). It requires consumers to perceive that the brand has a particular professional ability and reliability while consumers are willing to consume it (Rizvi et al., 2018). The establishment and maintenance of brand reputation are achieved through brand speculation and long-term marketing efforts (Keller, 2016). Furthermore, the brand investment represents the amount of money spent by the company on its brand. The higher significance of the investment further indicates that the company is willing to abide by its brand promise (Keller, 1993). Brand credibility is a long-term enterprise strategy, while a trustworthy brand establishes a long-term stable relationship with consumers (Baek et al., 2010). Brand credibility is an essential factor influencing brand equity (Ozdemir et al., 2020). The relationship between brand credibility and brand equity is significantly positive (Papasolomou and Vrontis, 2006). In the field of marketing, the effort represents the performance of enterprises. In green product marketing, establishing an eco-friendly corporate image largely depends on consumers’ trust in the brand. Therefore, brand credibility could positively impact green brand image (Leischnig et al., 2012). This study assumed that brand credibility would positively impact on brand equity.

Hypothesis (H8): *Brand credibility has a positive effect on brand equity*

### Attitude Toward Green Cosmetics

Under the influence of celebrity endorsement, consumers would form their attitudes toward certain products based on their preference and recognition of celebrities (Wymer and Drollinger, 2014). Knoll and Matthes (2016) postulated that the influence of celebrities on consumers’ attitude toward products refers to whether the consumers could accept celebrities’ endorsements and their level of interest toward endorsement advertisements. When consumers see their favorite stars endorsing a particular product, they would be more willing to buy the product (Myrick and Evans, 2014). Consumers’ behavior influenced by their purchase intention, which is affected by consumers’ attitudes toward the product (Ozdemir et al., 2020). According to Umberson’s (2008) study, the positive attitude toward green products often determines consumers’ purchase intention. Similarly, environmentally conscious attitudes directly or indirectly impact the purchase of green products (Nam et al., 2017).

Studies found a positive correlation between consumers’ attitudes toward celebrity endorsement and their products (Tantawi and Sadek, 2019). Consumers’ purchase intention refers to the consumption tendency of purchasing a specific product (Patel and Basil, 2017). According to the research, consumers’ positive attitudes toward celebrity endorsements will positively impact purchase intention (Roll, 2015). Therefore, based on the above literature review, this study hypothesized that consumers’ attitudes toward products as an impact from celebrity endorsement would positively impact on purchasing intention.

Hypothesis (H9): *The attitude toward green cosmetics has a positive impact on the intention to purchase green cosmetics*

### Brand Equity

Brand equity is vital in the field of corporate marketing. It was found that the increase in brand equity impacted retaining customers, increasing partners, and improving corporate profit margins (Park et al., 2019). From the perspective of enterprise management, enterprises could gain sustainable development by increasing brand equity (Rossiter, 2014). Meanwhile, consumer-level brand equity increased the likelihood of consumers’ choosing the brand, and broadened the channels to retain customers (Keller, 2018). Brand equity is defined as a brand name or brand symbol. In his research, brand loyalty, perceived quality, brand awareness, and brand correlation degree were the determinants used to examine brand equity and verify that these dimensions had significant positive effects on brand equity through the structural model (Sharma, 2017). The brand value of sportswear in the Chinese market is determined by brand awareness and perceived quality by Yoo et al. (2018).

Based on the past studies, brand loyalty, perceived quality, brand awareness, brand trust, and brand association identified the determinants of brand equity. According to the past research theories, the theoretical model of this study was determined (Keller, 2018). Brand equity was regarded as consumers’ cognition and familiarity with a brand. Therefore, brand equity could also represent the brand’s importance in the market (Kananukul et al., 2014). This study assumed that increased enterprise brand equity would positively impact consumers’ purchase intention.

Hypothesis (H10): *Brand equity has a positive on the intention to purchase green cosmetics*

### Mediating Effect of Attitude Toward Green Cosmetics

Tantawi and Sadek (2019) postulated a significant positive relationship between celebrity endorsement and consumers’ buying attitudes. It was proven in Thamaraiselvan et al.’s (2017) study that consumers’ purchase attitude would impact their buying behavior. Therefore, it was assumed that Chinese consumers’ attitudes toward green cosmetics would mediate the relationship between celebrity endorsement and intention to purchase green cosmetics as an intermediate variable for the current study.

Hypothesis (H11): *The attitude toward green cosmetics mediates the relationship between celebrity attractiveness, celebrity trustworthiness, and celebrity cause fit on the intention to purchase green cosmetics*

### Mediating Effect of Brand Equity

The current literature on brand equity determined that the structural model impacted brand equity. It was believed that brand awareness, brand loyalty, brand credibility, brand association, and perceived quality positively correlated with brand equity (Sharma, 2017). Therefore, brand equity was predicted to adjust the relationship between independent variables and purchase intention as an intermediate variable.

Hypothesis (H12): *Brand equity mediates the relationship between Brand Awareness, Brand Associations, Brand Loyalty, Perceived Quality, Brand Credibility on an intention to purchase green cosmetics*

**FIGURE 1:**
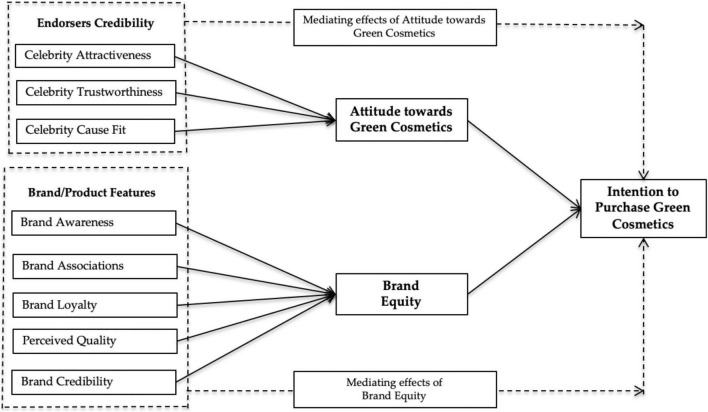
Research framework.

All association hypothesized and tested, presented in Figure 1.

## Research Methodology

### Research Design

The current paper aims to study the influencing factors of green cosmetics purchase by young consumers in China. Therefore, the research objects mainly consisted of young consumers in China. According to the nature of the study, a quantitative research design was used to study the relationship between various variables to test a theory or model. This study attempts to verify the relationship between celebrity endorsement, brand equity, and Chinese consumers’ intention to buy green cosmetics. Therefore, quantitative research was the most appropriate design method. The Study variables and questions were designed based on the previous relevant questionnaires. The questionnaires were distributed through social media (WeChat and other Internet platforms) to collect the respondents’ data with qualifying questions in COVID-19 time.

### Population and Sample

Following the increased concern about green consumption, people have begun to advocate the purchase of green products, which is accepted by many consumers worldwide. Therefore, this study mainly involved Chinese consumer groups and the factors influencing the willingness to purchase green cosmetics in China. The sampling survey method was adopted and distributed through WeChat, Weibo, and other Internet platforms. The probability sampling method applied in the sample target group ensured that everyone gained an equal opportunity to collect the questionnaire and ensure the questionnaire’s fairness and reliability (Sahu, 2016).

The research objects focused on young people aged 18 to 39 years old with a preference for trends and new things. Most of the target group gained income that could meet daily expenses. The target number of population samples to be collected was 300. The information collected by design mainly included basic issues, such as age, gender, monthly income, education level, and the monthly cost of buying cosmetics. The study results were summarized through the statistics of necessary population information.

### Survey Instrument

The research tool used in this study was a questionnaire. The first part of the survey questionnaire collected the interviewees’ basic information, including their gender, age, education level, monthly income, and other essential information. This information determined whether the respondents were the target population of this study or vice versa. The second part focused on the design of independent and dependent variables. Specifically, the dependent variables comprised celebrity endorsement and brand equity, which were used as independent variables to test the degree of influence on Chinese consumers’ willingness to buy green cosmetics (all questions and sources presented in Table 1). Following that, 1 to 5 options were set according to the degree of recognition (strongly disagree, disagree, neither agree nor disagree, agree and strongly agree). People who filled in the questionnaire could select from these options according to their level of recognition.

**TABLE 1:** Survey questionnaire.

Code	Questions	Sources
CA – Item 1	The celebrity endorser is attractive	Tantawi and Sadek, 2019
CA – Item 2	The celebrity endorser is classy.	
CA – Item 3	The celebrity endorser is elegant.	
CA – Item 4	The celebrity endorser is presentable.	
CA – Item 5	The celebrity endorser is well known	
CT – Item 1	The celebrity endorser is honest.	Tantawi and Sadek, 2019
CT – Item 2	The celebrity endorser is reliable.	
CT – Item 3	The celebrity endorser is trustworthy.	
CT – Item 4	The celebrity endorser is dependable.	
CT – Item 5	The celebrity endorser is sincere.	
CF – Item 1	It is very logical for the celebrity to endorse products	Thamaraiselvan et al., 2017
CF – Item 2	The celebrity appropriate to endorse the products is very important.	
CF – Item 3	The cause matched with celebrity personality.	
CF – Item 4	The celebrity and the product company should be represent to each other well.	
CF – Item 5	The celebrity and the product company should fit together well.	
AGC – Item 1	I like buying products which donate part of their profits to environment.	Thamaraiselvan et al., 2017
AGC – Item 2	I am willing to pay more for a product if the manufacturer donates part of the profits to environmental protection.	
AGC – Item 3	If a company is donating part of its profits to produce green products then I am more likely to buy its products.	
AGC – Item 4	Companies who advertise that they are donating part of their profits to environmental protection are good corporate citizens.	
AGC – Item 5	I make a special effort to buy from companies that support environmental causes.	
BA – Item 1	I can recognize green brand among other competing brands	Sharma, 2017
BA – Item 2	I am aware of green brand cosmetics	
BA – Item 3	The green brand is known to me.	
BA – Item 4	I am acquainted with the green brand cosmetics	
BA – Item 5	I know green brand cosmetics very well	
PQ – Item 1	Green cosmetics are high quality	Park et al., 2019
PQ – Item 2	The likely quality of green cosmetics is extremely high	
PQ – Item 3	The likelihood that green cosmetics is reliable is very high	
PQ – Item 4	The likelihood that green cosmetics will be satisfactory is very high	
PQ – Item 5	Green cosmetics is a quality leader within its category	
BL – Item 1	I consider myself to be loyal to green cosmetics	Sharma, 2017
BL – Item 2	I will not buy other brands if green cosmetics are available at the store	
BL – Item 3	Even if another product has the same features as the cosmetics, I would prefer to buy green cosmetics.	
BL – Item 4	Green cosmetics would be my first choice	
BL – Item 5	Green cosmetics are products that I often buy back	
BS – Item 1	You can recognize this brand among other competing brands because of its environmental commitments	Akturan, 2018
BS – Item 2	You are aware of this brand because of its environmental reputation	
BS – Item 3	Some environmental characteristics of this brand come to the top-of-mind in your consideration set quickly	
BS – Item 4	You can quickly recall the green image of this brand	
BS – Item 5	I have a clear impression of the type of people who use the green brand	
BC – Item 1	The green brand has the ability to deliver what it promises	Akturan, 2018
BC – Item 2	Green brand’s product claims are believable	
BC – Item 3	Over time, my experiences with the green brand have led me to expect it to keep its promises.	
BC – Item 4	The green brand has a name you can trust	
BC – Item 5	Green brands have their own techniques that you can trust	
BE – Item 1	It makes sense to buy this brand instead of other brands because of its environmental commitments, even if they are the same.	Sharma, 2017
BE – Item 2	Even if another brand has the same cosmetics features as green brand products, I would prefer to buy green brand products	
BE – Item 3	If there is another cosmetics performance greater than green cosmetics, I would prefer to buy green brand products	
BE – Item 4	If the performance concern of another brand is not different from that of this brand in any way, it seems smarter to purchase a green brand	
BE – Item 5	Buying green cosmetics is more attractive than other brands’ assets because it is in line with the concept of sustainable development	
IGC – Item 1	I intend to buy green cosmetics in the future	Nam et al., 2017
IGC – Item 2	I will try to buy green cosmetics in the future	
IGC – Item 3	I will make an effort to buy green cosmetics in the future	
IGC – Item 4	I would be willing to influence others to purchase green cosmetics-related products.	
IGC – Item 5	The likelihood of purchasing this product is very high	
IGC – Item 6	The probability that I would consider buying the product is very high	
IGC – Item 7	My willingness to buy the product is very high	

*CA: Celebrity Attractiveness, CT: Celebrity Trustworthiness, CF: Celebrity Cause Fit, AGC: Attitude Toward Green Cosmetics, BA: Brand Awareness, BS: Brand Associations, BL: Brand Loyalty, PQ: Perceived Quality, BC: Brand Credibility, BEQ: Brand Equity, IGC: Intention to Purchase Green Cosmetics.*

### Common Method Bias

Common method variance (CMV) is common in social science research and primarily generated due to a single source of data collection methods and techniques (Podsakoff et al., 2003). Hermans’s (1970) one-factor test was recommended to manage the CMV (Podsakoff et al., 2003). One-factor Harman’s test was applied to evaluate the issue of CMV in the study. It was found that no critical issue of CMV was present as the main factor accounted for 28.75% variance and was below the recommended limit of 50% (Podsakoff et al., 2003).

### Multivariate Normality

Peng and Lai (2012) suggested that general statements should not be made about the ability of the PLS estimation model. It could violate the multivariate normal assumption although PLS does not require a multivariate normal data distribution (Cain et al., 2017; Hair et al., 2019). This study used the Smart-PLS online tool to test multivariate normality. According to the calculation, it was found that the *p*-Values of the multivariate skewness and kurtosis coefficient of Mardias were lower than 0.05, proving the irregular current data.

### Data Analysis Method

The results from the analysis of the collected data using SmartPLS software summarized the study conclusions and insights. The primary contents included (1) basic description analysis, mainly used to describe the central trend and quantitative data distribution, (2) reliability analysis, (3) validity analysis mainly including convergence validity and discriminant validity, and (4) influence relationship study (Hair et al., 2019). Cronbach’s Alpha reliability coefficient method is the most commonly used. Cronbach’s alpha’s reliability coefficient was tested to measure whether the research data reached the standard or vice versa. Convergent validity validation factor analysis of AVE, CR, and other indicators made a comparison between validity validation AVE and correlation analysis results (Chin, 2010). The study focused on data path analysis to study the relationship between multiple independent variables (Hair et al., 2019).

## Research Findings

### Demographic Details

Table 2 presents the demographic data of 301 respondents, including five questions. Based on this sample survey, 248 female respondents accounted for 82.4%, while 17.6% were male. This paper mainly focuses on research on the purchase of green cosmetics among Chinese women. In launching the questionnaire, the target group was mainly female, while the proportion of male interviewees was significantly lower compared to female interviewees. Most (37.5%) of the respondents were aged between 18 and 25. As a result, more young people were surveyed. The remaining groups aged 26–30 and 31–40 years old, 34.2% and 22.9%, respectively. In terms of income, most of the interviewees gained a monthly income of 1000–3000 (RMB), which accounted for 32.9%. The second group consisted of interviewees with a monthly income of 3000–5000 (RMB), which accounted for 26.6% of the respondents. Most of the respondents have bachelor’s degrees (41.5%). According to the data results, most young Chinese consumers spend less than 500 yuan (RMB) per month on green cosmetics. The interviewed group was relatively young, mainly consisted of students and the starting phase of social work.

**TABLE 2:** Demographic characteristics.

	N	%		N	%
**Gender**			**Education**		
Female	248	82.4	junior high school	5	1.7
Male	53	17.6	high school	10	3.3
Total	301	100.0	Diploma	104	34.6
			Bachelor Degree	125	41.5
**Age Group**			Master Degree	52	17.3
18 – 25 years old	113	37.5	Doctorate Degree	5	1.7
26 – 30 years old	104	34.6	Total	301	100.0
31 – 40 years old	69	22.9			
41 – 50 years old	11	3.7	**Average Monthly Income (Yuan)**		
>51 years old	4	1.3	Less than 1000 Yuan	44	14.6
Total	301	100.0	1001–3000 Yuan	99	32.9
			3001–5000 Yuan	80	26.6
**Monthly Budget for Cosmetics (Yuan)**			5001–7000 Yuan	27	9.0
Less than 500 Yuan	148	49.2	7001–9000 Yuan	21	7.0
501–1500 Yuan	90	29.9	9001–10000 Yuan	18	6.0
1501–3000 Yuan	54	17.9	More than 10001 Yuan	12	4.0
More than 3001 Yuan	9	3.0	Total	301	100.0
Total	301	100.0			

### Reliability and Validity

Table 3 illustrates the reliability of each item in this study, which mainly refers to the reliability of the research data results. All variables’ mean and standard deviation (celebrity attractiveness, celebrity trustworthiness, celebrity cause fit, attitude toward green cosmetics, brand awareness, brand associations, brand loyalty, perceived quality, brand credibility, brand equity, intention to purchase green cosmetics) are presented in Table 3. According to the data results, the mean value of celebrity trustworthiness, brand awareness, and deviation of celebrity trustworthiness are relatively small. This result indicated that most young consumers do not believe that celebrity endorsement has integrity or celebrity credibility will not improve Chinese consumers’ attitudes toward green cosmetics.

**TABLE 3:** Reliability and validity.

Variables	No. Items	Mean	*SD*	CA	DG *rho*	CR	AVE	VIF
CA	5	3.447	0.844	0.880	0.887	0.912	0.676	1.801
CT	5	2.746	0.841	0.889	0.891	0.919	0.693	1.272
CF	5	3.320	0.926	0.944	0.945	0.957	0.818	1.823
AGC	5	3.483	0.897	0.919	0.920	0.939	0.756	1.392
BA	5	2.870	0.996	0.937	0.942	0.952	0.799	1.796
BS	5	3.127	0.929	0.956	0.958	0.966	0.852	2.878
BL	5	3.271	0.967	0.947	0.949	0.959	0.826	1.601
PQ	5	2.940	0.962	0.951	0.952	0.962	0.835	1.877
BC	5	2.993	0.981	0.954	0.955	0.965	0.845	2.248
BEQ	5	3.290	0.923	0.941	0.942	0.955	0.811	1.392
IGC	7	3.512	0.992	0.971	0.972	0.976	0.853	-

*CA: Celebrity Attractiveness, CT: Celebrity Trustworthiness, CF: Celebrity Cause Fit, AGC: Attitude Toward Green Cosmetics, BA: Brand Awareness, BS: Brand Associations, BL: Brand Loyalty, PQ: Perceived Quality, BC: Brand Credibility, BEQ: Brand Equity, IGC: Intention to Purchase Green Cosmetics, SD: Standard Deviation, CAA: Cronbach’s Alpha, DG rho: Dillon-Goldstein’s rho, CR: Composite Reliability, AVE: Average Variance Extracted, VIF: Variance Inflation Factors. Author’s data analysis.*

Cronbach’s Alpha was used to measure the reliability of data results. The data could be considered reliable with a value of >0.7. The value reliability ranged from 0 to 1, with a more reliable value achieved when the value was closer to 1. According to the data results, the Cronbach’s Alpha value of each variable was higher than 0.8, leading to a high degree of reliability. Generally, the requirement for Dillon Goldstein’s rho value is >0.65, which was fulfilled by questionnaire results. Furthermore, AVE indicates the level of representation of the dependent variable made through each independent variable. The AVE value of >0.5 indicated good convergence, which was in line with the data from the analysis results. VIF is an index to measure the degree of collinearity in multiple linear regression models. A higher value of VIF increased the possibility of commonality between variables. Generally, the value of VIF ranges from 0.1 to 10, indicating the absence of collinearity issue between the variables. Therefore, the VIF results of this study were within a reasonable range.

According to the discriminant validity principle of the Fornell-Larcker Criterion, the AVE square root value of the dimension should be higher compared to the correlation coefficient below that value. As shown in Table 4, the AVE square root of the data result dimension was significantly higher than the coefficient results below. Therefore, the data were in line with the criteria. Furthermore, HTMT refers to the mean value of the correlation between the indexes of different dimensions and the mean value of indexes of the same dimension. With a similar concept of variables in the dimension, the threshold value of HTMT was maintained at 0.9. According to the calculation results, given that the HTMT data result amounted to 0.876 < 0.9, the research data fulfilled the standard.

**TABLE 4:** Discriminant validity.

	CA	CT	CF	AGC	BA	BS	BL	PQ	BC	BEQ	IGC
**Fornell-Larcker Criterion**											
CA	0.822										
CT	0.414	0.833									
CF	0.649	0.426	0.905								
AGC	0.642	0.423	0.657	0.869							
BA	0.444	0.493	0.444	0.457	0.894						
BS	0.479	0.537	0.483	0.535	0.638	0.923					
BL	0.550	0.341	0.461	0.595	0.454	0.575	0.909				
PQ	0.388	0.536	0.358	0.401	0.514	0.577	0.489	0.914			
BC	0.323	0.535	0.322	0.369	0.494	0.702	0.419	0.607	0.919		
BEQ	0.506	0.410	0.503	0.531	0.446	0.573	0.571	0.538	0.602	0.900	
IGC	0.588	0.298	0.550	0.540	0.406	0.435	0.553	0.454	0.352	0.553	0.923
**Heterotrait-Monotrait Ratio (HTMT)**											
CA	–										
CT	0.476	–									
CF	0.707	0.463	–								
AGC	0.708	0.467	0.702	–							
BA	0.485	0.538	0.471	0.485	–						
BS	0.528	0.583	0.506	0.570	0.671	–					
BL	0.605	0.372	0.486	0.638	0.479	0.603	–				
PQ	0.428	0.583	0.376	0.429	0.542	0.605	0.514	–			
BC	0.358	0.580	0.339	0.395	0.524	0.735	0.439	0.637	–		
BEQ	0.555	0.448	0.532	0.571	0.469	0.603	0.603	0.568	0.635	–	
IGC	0.630	0.320	0.573	0.571	0.419	0.451	0.576	0.471	0.365	0.577	–

*CA: Celebrity Attractiveness, CT: Celebrity Trustworthiness, CF: Celebrity Cause Fit, AGC: Attitude Toward Green Cosmetics, BA: Brand Awareness, BS: Brand Associations, BL: Brand Loyalty, PQ: Perceived Quality, BC: Brand Credibility, BEQ: Brand Equity, IGC: Intention to Purchase Green Cosmetics.*

*Author’s data analysis.*

Table 5 illustrates the value of cross-loading, reflecting the comparison between the load value and the value of cross-loading. According to the data results, the load value was higher compared to the cross-loading value. Besides, each item load value was higher than 0.7, proving discriminant validity in the data.

**TABLE 5:** Loadings and cross-loading.

Code	CA	CT	CF	AGC	BA	BS	BL	PQ	BC	BEQ	IGC
CA – Item 1	0.814	0.272	0.523	0.531	0.348	0.345	0.393	0.272	0.231	0.393	0.480
CA – Item 2	0.760	0.430	0.445	0.421	0.358	0.448	0.463	0.356	0.324	0.377	0.374
CA – Item 3	0.843	0.417	0.509	0.537	0.391	0.465	0.488	0.326	0.305	0.448	0.474
CA – Item 4	0.834	0.330	0.588	0.543	0.384	0.386	0.471	0.341	0.275	0.425	0.524
CA – Item 5	0.857	0.282	0.588	0.586	0.348	0.348	0.454	0.315	0.214	0.431	0.545
CT – Item 1	0.344	0.815	0.335	0.337	0.368	0.439	0.291	0.478	0.497	0.302	0.188
CT – Item 2	0.305	0.845	0.343	0.346	0.384	0.469	0.289	0.439	0.457	0.339	0.249
CT – Item 3	0.369	0.804	0.357	0.332	0.433	0.457	0.276	0.407	0.398	0.351	0.297
CT – Item 4	0.316	0.861	0.346	0.362	0.392	0.431	0.269	0.483	0.433	0.381	0.252
CT – Item 5	0.388	0.837	0.389	0.380	0.473	0.440	0.294	0.427	0.444	0.334	0.254
CF – Item 1	0.591	0.449	0.833	0.615	0.425	0.480	0.454	0.400	0.375	0.524	0.540
CF – Item 2	0.572	0.371	0.908	0.547	0.406	0.417	0.374	0.304	0.277	0.418	0.478
CF – Item 3	0.587	0.378	0.927	0.604	0.397	0.408	0.395	0.292	0.240	0.429	0.480
CF – Item 4	0.585	0.348	0.930	0.593	0.396	0.425	0.431	0.298	0.264	0.437	0.480
CF – Item 5	0.598	0.374	0.922	0.603	0.381	0.447	0.424	0.318	0.295	0.457	0.502
AGC – Item 1	0.578	0.382	0.642	0.856	0.401	0.437	0.497	0.328	0.299	0.452	0.450
AGC – Item 2	0.572	0.401	0.555	0.890	0.388	0.459	0.504	0.381	0.306	0.468	0.475
AGC – Item 3	0.560	0.341	0.567	0.884	0.389	0.428	0.524	0.331	0.312	0.449	0.481
AGC – Item 4	0.545	0.351	0.576	0.866	0.364	0.454	0.523	0.328	0.314	0.460	0.452
AGC – Item 5	0.532	0.363	0.510	0.850	0.443	0.548	0.542	0.374	0.377	0.477	0.488
BA – Item 1	0.450	0.449	0.382	0.486	0.825	0.582	0.437	0.472	0.428	0.467	0.430
BA – Item 2	0.392	0.435	0.408	0.398	0.925	0.562	0.407	0.420	0.417	0.365	0.355
BA – Item 3	0.384	0.429	0.424	0.412	0.934	0.559	0.381	0.428	0.432	0.391	0.355
BA – Item 4	0.340	0.469	0.360	0.310	0.845	0.573	0.392	0.522	0.514	0.356	0.282
BA – Item 5	0.393	0.413	0.403	0.402	0.936	0.562	0.399	0.446	0.416	0.387	0.365
BS – Item 1	0.459	0.471	0.466	0.544	0.591	0.902	0.524	0.524	0.647	0.569	0.403
BS – Item 2	0.452	0.489	0.444	0.501	0.598	0.929	0.517	0.535	0.627	0.497	0.394
BS – Item 3	0.439	0.497	0.434	0.480	0.553	0.925	0.517	0.511	0.653	0.518	0.403
BS – Item 4	0.433	0.472	0.456	0.477	0.610	0.949	0.546	0.545	0.670	0.533	0.412
BS – Item 5	0.427	0.548	0.422	0.460	0.589	0.909	0.549	0.547	0.637	0.522	0.393
BL – Item 1	0.490	0.295	0.425	0.531	0.437	0.529	0.928	0.451	0.387	0.530	0.502
BL – Item 2	0.496	0.307	0.420	0.535	0.363	0.447	0.864	0.391	0.306	0.502	0.505
BL – Item 3	0.504	0.294	0.431	0.550	0.407	0.571	0.902	0.478	0.430	0.554	0.510
BL – Item 4	0.489	0.320	0.390	0.515	0.418	0.517	0.916	0.451	0.380	0.476	0.489
BL – Item 5	0.519	0.334	0.425	0.570	0.437	0.544	0.930	0.445	0.394	0.523	0.502
PQ – Item 1	0.338	0.507	0.312	0.366	0.482	0.531	0.439	0.931	0.560	0.482	0.386
PQ – Item 2	0.352	0.465	0.305	0.378	0.445	0.512	0.415	0.886	0.539	0.454	0.393
PQ – Item 3	0.363	0.479	0.349	0.362	0.476	0.539	0.466	0.908	0.562	0.516	0.443
PQ – Item 4	0.347	0.502	0.335	0.366	0.481	0.526	0.457	0.922	0.550	0.502	0.424
PQ – Item 5	0.374	0.498	0.330	0.360	0.463	0.528	0.452	0.922	0.561	0.502	0.424
BC – Item 1	0.328	0.541	0.322	0.381	0.456	0.680	0.428	0.572	0.937	0.579	0.340
BC – Item 2	0.306	0.485	0.264	0.329	0.429	0.635	0.425	0.559	0.903	0.556	0.339
BC – Item 3	0.299	0.490	0.283	0.313	0.460	0.616	0.350	0.563	0.932	0.562	0.299
BC – Item 4	0.267	0.481	0.295	0.334	0.439	0.639	0.352	0.554	0.946	0.553	0.298
BC – Item 5	0.285	0.457	0.316	0.341	0.489	0.656	0.370	0.540	0.875	0.514	0.342
BEQ – Item 1	0.478	0.363	0.487	0.494	0.406	0.532	0.487	0.487	0.558	0.890	0.470
BEQ – Item 2	0.459	0.390	0.452	0.480	0.410	0.529	0.503	0.504	0.583	0.931	0.513
BEQ – Item 3	0.418	0.412	0.398	0.437	0.432	0.493	0.519	0.499	0.553	0.901	0.481
BEQ – Item 4	0.484	0.322	0.503	0.519	0.395	0.508	0.545	0.466	0.469	0.868	0.519
BEQ – Item 5	0.437	0.359	0.425	0.459	0.366	0.520	0.516	0.465	0.546	0.911	0.504
IGC – Item 1	0.556	0.284	0.511	0.517	0.385	0.425	0.546	0.419	0.332	0.512	0.937
IGC – Item 2	0.581	0.287	0.538	0.517	0.394	0.425	0.506	0.437	0.363	0.525	0.900
IGC – Item 3	0.528	0.240	0.505	0.493	0.373	0.398	0.520	0.405	0.302	0.499	0.936
IGC – Item 4	0.522	0.248	0.502	0.473	0.378	0.383	0.490	0.410	0.297	0.491	0.932
IGC – Item 5	0.551	0.315	0.506	0.482	0.391	0.432	0.538	0.486	0.356	0.502	0.914
IGC – Item 6	0.523	0.273	0.494	0.484	0.317	0.360	0.460	0.372	0.297	0.494	0.910
IGC – Item 7	0.539	0.276	0.495	0.518	0.383	0.386	0.510	0.402	0.323	0.545	0.933

*(1) NFA: Need for Achievement, RTP: Risk-Taking Propensity, PRP: Proactive Personality, SLE: Self-Efficacy, ENE: Entrepreneurship Education, UNA: Uncertainty Avoidance, ENK: Entrepreneurial Knowledge, ATE: Attitude Toward Entrepreneurship, ORC: Opportunity Recognition Competency, ENIN: Entrepreneurial Intention. (2) The Italic values in the matrix above are the item loadings and others are cross-loadings.*

*Author’s data analysis.*

### Path Analysis

As shown in Table 6, the path coefficients demonstrated the coefficient value for celebrity attractiveness on consumers’ attitude toward green cosmetics (β = 0.345, *p*-Value = 0.000), which indicated that celebrity attractiveness had a significantly positive effect on consumers’ attitude toward green cosmetics. The f^2^ value of 0.138 represented a small effect size (Cohen, 2013). Meanwhile, the coefficient value for celebrity trustworthiness on consumers’ attitudes toward green cosmetics (β = 0.117, *p*-Value = 0.003) indicated that celebrity trustworthiness significantly affecting consumers’ attitudes toward green cosmetics. Following that, the f^2^ value of 0.023 showed a small effect size (Cohen, 2013). The coefficient value for celebrity cause fit on consumers’ attitude toward green cosmetics (β = 0.383, *p*-Value = 0.000) implied that celebrity cause fit had a significantly positive effect on consumers’ attitude toward green cosmetics. The f^2^ value of 0.168 denoted a small media effect size (Cohen, 2013). Moreover, the r^2^ value, which represented the degree to which the independent variable explained the dependent variable, amounted to 0.522, indicating that a significant proportion of 52.2% of the variation in consumers’ attitude toward green cosmetics could be represented by the level of celebrity attractiveness, celebrity trustworthiness, and celebrity cause fit (Hair et al., 2019). The Q^2^ value of 0.387 indicated that consumers’ levels of celebrity attractiveness, celebrity trustworthiness, and celebrity cause fit have a large predictive relevance for their attitudes toward green cosmetics among young Chinese (Cohen, 2013).

**TABLE 6:** Path coefficients.

Hypo		Beta	CI – Min	CI – Max	*t*	*p*	*r* ^2^	*f* ^2^	Q^2^	Decision
**Factors Affecting Attitude Toward Green Cosmetics**										
H_1_	CA→AGC	0.345	0.242	0.442	5.806	0.000		0.138		Accept
H_2_	CT→AGC	0.117	0.051	0.192	2.794	0.003	0.522	0.023	0.387	Accept
H_3_	CF→AGC	0.383	0.288	0.472	6.997	0.000		0.168		Accept
**Factors Affecting Brand Equity**										
H_4_	BA→BEQ	0.031	–0.065	0.137	0.498	0.310		0.001		Reject
H_5_	BS→BEQ	0.065	–0.096	0.208	0.732	0.232		0.003		Reject
H_6_	BL→BEQ	0.318	0.218	0.431	5.101	0.000		0.126		Accept
H_7_	PQ→BEQ	0.130	0.007	0.251	1.663	0.048	0.500	0.018	0.400	Accept
H_8_	BC→BEQ	0.329	0.173	0.475	3.612	0.000		0.097		Accept
**Factor Affecting Intention to Purchase Green Cosmetics**										
H_8_	AGC→IGC	0.343	0.245	0.439	5.903	0.000	0.390	0.138	0.329	Accept
H_9_	BEQ→IGC	0.371	0.259	0.463	6.008	0.000		0.162		Accept

*CA: Celebrity Attractiveness, CT: Celebrity Trustworthiness, CF: Celebrity Cause Fit, AGC: Attitude Toward Green Cosmetics, BA: Brand Awareness, BS: Brand Associations, BL: Brand Loyalty, PQ: Perceived Quality, BC: Brand Credibility, BEQ: Brand Equity, IGC: Intention to Purchase Green Cosmetics.*

*Author’s data analysis.*

The coefficient value for brand awareness on brand equity (β = 0.031, *p*-Value = 0.310) indicated that brand awareness had no significant impact on brand equity. The f^2^ value of 0.001 denoted the absence of effect size and predictive relevance of brand awareness for brand equity (Cohen, 2013). The coefficient value for brand associations on brand equity (β = 0.065, *p*-Value = 0.232) demonstrated that brand associations had no significant effect on brand equity. Furthermore, the f^2^ value of 0.003 suggested no effect size and brand association predictive relevance for brand equity (Cohen, 2013). The path coefficient value for brand loyalty on brand equity (β = 0.318, *p*-Value = 0.000) indicated that brand loyalty had a significantly positive effect on brand equity, while the f^2^ value of 0.126 represented a small effect size (Cohen, 2013). The perceived quality coefficient shows a positive (β = 0.130, *p*-Value = 0.045) effect on brand equity. Moreover, the f^2^ value of 0.018 showed a small effect size of perceived quality for brand equity (Cohen, 2013). The path coefficient value for brand credibility on brand equity (β = 0.329, *p*-Value = 0.00) indicated that brand credibility had a significantly positive effect on brand equity, while the f^2^ value of 0.097 indicated a small effect size (Cohen, 2013). The r^2^ value, which represented the degree of explained variance, amounted to 0.5, indicating that a significant proportion (50%) of the variation in brand equity could be illustrated through brand awareness, brand associations, brand loyalty, perceived quality, and brand credibility (Hair et al., 2019). On the other hand, the Q^2^ value of 0.4 indicated high predictive relevance of brand awareness, brand associations, brand loyalty, perceived quality, and brand credibility for brand equity (Cohen, 2013).

The coefficients for attitude toward green cosmetics showed a positive effect on the intention to purchase green cosmetics (β = 0.343, *p*-Value = 0.000). The f^2^ value of 0.138 illustrated a small to the regular effect of attitude toward green cosmetics on the intent to purchase green cosmetics (Cohen, 2013). The coefficient for brand equity on the intention to purchase green cosmetics (β = 0.371, *p*-Value = 0.000) indicated that brand equity had a significantly positive effect on the intention to purchase green cosmetics. The f^2^ value of 0.162 denoted the size of the media effect (Cohen, 2013). Furthermore, the r^2^ value, which represented the degree of explained variance, amounted to 0.39, indicating that a significant proportion (39%) of the variation in the intention to purchase green cosmetics was represented by the attitude toward green cosmetics and brand equity (Hair et al., 2019). The Q^2^ value of 0.329 demonstrated a large predictive relevance of attitude toward green cosmetics and brand equity to purchase green cosmetics among young Chinese (Cohen, 2013).

### Mediating Effects

Regarding the mediating effects of attitudes on green cosmetics and brand equity, the study presented the indirect effects and *p*-Values in Table 7. It was found that celebrity’s attractiveness, trustworthiness, and cause-fit had a significantly (*p*-Values < 0.05) positive indirect effect on the intention to purchase green cosmetics among young Chinese consumers, confirming the mediating effect of attitude toward green cosmetics. Furthermore, brand loyalty and brand credibility had a significant (*p*-Value < 0.05) positive indirect effect on young Chinese consumers’ intention to purchase green cosmetics, confirming the mediating effect of brand equity.

**TABLE 7:** Mediating effects.

Associations	Beta	CI – Min	CI – Max	*T*	*p*	Decision
**Mediating effect of Attitude Toward Green Cosmetics**						
CA→AGC→IGC	0.118	0.069	0.175	3.594	0.000	Accept
CT→AGC→IGC	0.040	0.019	0.069	2.658	0.004	Accept
CF→AGC→IGC	0.131	0.087	0.179	4.760	0.000	Accept
**Mediating effect of Brand Equity**						
BA→BEQ→IGC	0.012	–0.027	0.050	0.494	0.311	Reject
BS→BEQ→IGC	0.024	–0.036	0.078	0.729	0.233	Reject
BL→BEQ→IGC	0.118	0.069	0.178	3.486	0.000	Accept
PQ→BEQ→IGC	0.048	0.003	0.098	1.597	0.055	Reject
BC→BEQ→IGC	0.122	0.063	0.189	3.162	0.001	Accept

*CA: Celebrity Attractiveness, CT: Celebrity Trustworthiness, CF: Celebrity Cause Fit, AGC: Attitude Toward Green Cosmetics, BA: Brand Awareness, BS: Brand Associations, BL: Brand Loyalty, PQ: Perceived Quality, BC: Brand Credibility, BEQ: Brand Equity, IGC: Intention to Purchase Green Cosmetics.*

*Author’s data analysis.*

### Predictive Assessment

The predictive power of the model was estimated with the PLS_*Predict*_. The assessment established the performance of the PLS model with new predictive observations. It was found that the paths exhibited high predictive relevance as the Q^2^_*predict*_ value was above 0 and 0.3 (Shumeli et al., 2019). The study assessed the predictive power based on comparing root mean squared error (RMSE) values from the PLS-SEM analysis and the LM benchmark. As presented in Table 8, the study model possessed high predictive power (Shumeli et al., 2019).

**TABLE 8:** Predictive model assessment.

	Q^2^_*Predict*_	RMSE (PLS-SEM)	RMSE (LM)	Difference	Predictive Power
AGC – Item 1	0.447	0.791	0.826	–0.034	
AGC – Item 2	0.389	0.788	0.812	–0.024	
AGC – Item 3	0.377	0.815	0.865	–0.050	High Predictive Power
AGC – Item 4	0.378	0.792	0.833	–0.042	
AGC – Item 5	0.328	0.870	0.850	0.019	
BEQ – Item 1	0.381	0.782	0.819	–0.036	
BEQ – Item 2	0.407	0.801	0.843	–0.042	
BEQ – Item 3	0.395	0.778	0.826	–0.048	High Predictive Power
BEQ – Item 4	0.356	0.832	0.871	–0.039	
BEQ – Item 5	0.381	0.837	0.881	–0.044	
IGC – Item 2	0.344	0.859	0.871	–0.012	
IGC – Item 3	0.349	0.867	0.896	–0.029	
IGC – Item 4	0.317	0.912	0.982	–0.071	
IGC – Item 5	0.307	0.897	0.936	–0.039	High Predictive Power
IGC – Item 6	0.350	0.861	0.891	–0.029	
IGC – Item 7	0.293	0.879	0.898	–0.018	
IGC – Item 2	0.320	0.911	0.971	–0.060	

*AGC: Attitude Toward Green Cosmetics, BA: Brand Awareness, BS: Brand Associations, BL: Brand Loyalty, PQ: Perceived Quality, BC: Brand Credibility, BEQ: Brand Equity, IGC: Intention to Purchase Green Cosmetics, MAE: Mean Absolute Error, RMSE: Root Mean Squared Error, PLS-SEM: Partial Least Squares – Structural Equation Modeling, LM: Linear Regression Model.*

*Author’s data analysis.*

## Discussion

The study confirmed the impact of celebrity endorsement and brand equity on young Chinese intention to buy green cosmetics. The influence of three dimensions of celebrity endorsement (celebrity attractiveness, celebrity trustworthiness, and celebrity cause fit) on consumers’ buying attitude and five dimensions of brand equity (brand awareness, brand credibility, brand associations, perceived quality, and brand loyalty) on green brands were examined. According to the research results, celebrity attractiveness, celebrity trustworthiness, and celebrity cause fit had significant positive effects on consumers’ attitudes toward green cosmetics in the dimension of celebrity endorsement, which was in line with the research results expressed by Thamaraiselvan et al. (2017). It was supported by the current study results where celebrity cause fit had the most substantial impact on consumer attitudes, which was in line with Akturan’s (2018) results that green product attributes affect green brand equity. The research results demonstrated that brand loyalty, brand credibility, and perceived quality had significant positive effects on brand equity for green cosmetics products among the Chinese youth. However, the study depicts that brand awareness and association did not significantly influence consumer brand equity. The study finding suggests that the low brand awareness among the study respondents leads to insignificant consumers’ level of brand equity. Our study finding matches the result postulated by Thamaraiselvan et al. (2017) that low consumer awareness cannot nurture brand equity among consumers. Furthermore, the brand association for Chinese consumers was not much built and unable to significantly instigates brand equity. The current finding contradicts the result posted by Tantawi and Sadek (2019), that the new fashion brand takes time to recall by the consumers and consumers unable to remember or cognize the brand. Therefore, the green cosmetic brand’s brand awareness was low and yielded low brand equity.

In addition, according to the data analysis, it was believed that consumers’ attitude toward green products has a significant positive impact on purchasing intention, which was consistent with the research findings postulated by Thamaraiselvan et al. (2017). Although brand equity had a significant positive effect on purchase intention, this argument was in line with the research conclusion drawn by Nam et al. (2017).

The mediating effect results demonstrated that celebrity attractiveness and celebrity cause fit had a significant positive impact on the intention of young Chinese consumers. Consumer’s attitude toward green cosmetics indicated that the purchasing attitude, as an intermediate variable, plays a particular mediating role between celebrity endorsement and purchase intention. Besides, green brand equity plays an intermediary role in brand loyalty and brand credibility. From the perspective of importance, consumers’ attitudes toward green cosmetics and green brand equity have the most significant impact on purchase intention, proving the positive impact of consumers’ attitudes and brand equity. In this study, the influence factors of celebrity endorsement and brand equity were combined to determine young Chinese consumers’ influence on their intention to buy green cosmetics, which further extended the previous research conclusions drawn with the new research results.

## Implications

Compared to the previous research theories, most of the studies only independently verified the impact of celebrity endorsement on purchase intention or independently verified the dimension of brand equity. The current study combined two significant celebrity endorsement and brand equity factors and targeted young consumers in the Chinese market who purchased green cosmetics as the research object. The influence of celebrity endorsement (celebrity attractiveness, celebrity trustworthiness, and celebrity cause fit) and green brand equity (brand awareness, brand credibility, brand associations, perceived quality, and brand loyalty) on the intention to buy green cosmetics was investigated. The following research results were recorded by setting the theoretical framework, presenting the hypotheses, and conducting data analysis. Celebrity attractiveness, celebrity trustworthiness, and celebrity cause fit significantly influenced consumers’ treatment of green cosmetics. Brand credibility, perceived quality, and brand loyalty significantly impacted green brand equity. Besides, celebrity attractiveness, celebrity cause fit, brand credibility, and brand loyalty significantly influenced buying green cosmetics under the influence of the two intermediary variables of purchase attitude and brand equity. These results enriched the literature on the purchase intention of green cosmetics and assisted in continuing other studies on the purchase intention of green consumer goods.

The results of this study also offered practical implications for brand owners. Brand owners could improve green cosmetics sales from the following aspects. Specifically, attractive celebrities, and celebrities’ suitability with the product image could be targeted (Arora et al., 2019). This type of celebrity endorsement creates more publicity effects for consumers to attract consumers’ attention and promote the sales of green cosmetics through celebrity endorsement advertising. On the other hand, green cosmetic enterprises should pay attention to improving brand loyalty and credibility (Beatson et al., 2020). According to the current study, upon purchasing products by consumers, the majority of them would be affected by brand loyalty and credibility. Therefore, the brand should improve customer satisfaction and form customer loyalty while continuously building an honest and trustworthy corporate image. However, false propaganda to deceive consumers is not a proper method to promote sales faster and improve brand value and corporate assets.

## Conclusion

This study aims to verify the influence of celebrity endorsement and brand equity on the intention of young Chinese to buy green cosmetics. It was found that celebrity attractiveness, celebrity cause fit, brand credibility, and brand loyalty significantly impacted purchase intention. This study presented a specific guiding significance in theory and practice. A theoretical model is established from an academic perspective by organizing various literature and synthesizing other research conclusions. The research results were obtained, providing a theoretical basis for the subsequent research on other purchase intentions for green consumer goods. From the perspective of practice, the investigation, and analysis of the market offer guidance and suggestions for green cosmetics enterprises to improve brand competitiveness and sales.

Certain limitations were present in this study as it mainly investigated the Chinese market, with the interviewees primarily originated from the north of China. When the conclusion of this study should be drawn in a broader scope, the research scope and replication studies should be performed in different cities to obtain more accurate results. Last but not least, following the principle of caution when interpreting this study’s results is essential. Besides, researching different countries or regions may lead to different results of this study.

## Data Availability Statement

The original contributions presented in the study are included in the article/Supplementary Material, further inquiries can be directed to the corresponding author/s.

## Ethics Statement

Ethical review and approval was not required for the study on human participants in accordance with the local legislation and institutional requirements. The patients/participants provided their written informed consent to participate in this study.

## Author Contributions

ZL, NH, QY, and AS did the conceptualization, performed the methodology, collected the data, and wrote the original draft. AM and MA performed the methodology (instrument), carried out the data analysis and wrote, edited, and revised the manuscript. All authors contributed to the article and approved the submitted version.

## Conflict of Interest

The authors declare that the research was conducted in the absence of any commercial or financial relationships that could be construed as a potential conflict of interest.

## Publisher’s Note

All claims expressed in this article are solely those of the authors and do not necessarily represent those of their affiliated organizations, or those of the publisher, the editors and the reviewers. Any product that may be evaluated in this article, or claim that may be made by its manufacturer, is not guaranteed or endorsed by the publisher.
